# RF EMF Risk Perception Revisited: Is the Focus on Concern Sufficient for Risk Perception Studies?

**DOI:** 10.3390/ijerph14060620

**Published:** 2017-06-08

**Authors:** Peter M. Wiedemann, Frederik Freudenstein, Christoph Böhmert, Joe Wiart, Rodney J. Croft

**Affiliations:** 1 School of Psychology, University of Wollongong, Northfields Ave, Wollongong, NSW 2522, Australia; peter.wiedemann@wf-emf.org (P.M.W.); rcroft@uow.edu.au (R.J.C.); 2 Illawarra Health and Medical Research Institute, University of Wollongong Campus, NSW 2522, Australia; 3 Australian Centre for Electromagnetic Bioeffects Research, NH&MRC Centre of Research Excellence, University of Wollongong, Wollongong, NSW 2522, Australia; 4 Centre for Population Population Health Research on Electromagnetic Energy, Monash University, Clayton, VIC 3800, Australia; 5 Department of Science Communication, Karlsruhe Institute of Technology, Englerstr. 2 76131 Karlsruhe, Germany; christoph.boehmert@kit.edu; 6 Chaire C2M, LTCI Telecom ParisTech, Paris Saclay University, 46 Rue Barrault, 75013 Paris, France; joe.wiart@mines.org

**Keywords:** RF EMF, exposure perception, risk perception, risk communication, base stations, mobile phones, questionnaire design, survey methodology, thematic relevance

## Abstract

An implicit assumption of risk perception studies is that concerns expressed in questionnaires reflect concerns in everyday life. The aim of the present study is to check this assumption, i.e., the extrapolability of risk perceptions expressed in a survey, to risk perceptions in everyday life. To that end, risk perceptions were measured by a multidimensional approach. In addition to the traditional focus on measuring the magnitude of risk perceptions, the thematic relevance (how often people think about a risk issue) and the discursive relevance (how often people think about or discuss a risk issue) of risk perceptions were also collected. Taking into account this extended view of risk perception, an online survey was conducted in six European countries with 2454 respondents, referring to radio frequency electromagnetic field (RF EMF) risk potentials from base stations, and access points, such as WiFi routers and cell phones. The findings reveal that the present study’s multidimensional approach to measuring risk perception provides a more differentiated understanding of RF EMF risk perception. High levels of concerns expressed in questionnaires do not automatically imply that these concerns are thematically relevant in everyday life. We use thematic relevance to distinguish between enduringly concerned (high concern according to both questionnaire and thematic relevance) and not enduringly concerned participants (high concern according to questionnaire but no thematic relevance). Furthermore, we provide data for the empirical value of this distinction: Compared to other participants, enduringly concerned subjects consider radio frequency electromagnetic field exposure to a greater extent as a moral and affective issue. They also see themselves as highly exposed to radio frequency electromagnetic fields. However, despite these differences, subjects with high levels of thematic relevance are nevertheless sensitive to exposure reduction as a means for improving the acceptance of base stations in their neighborhood. This underlines the value of exposure reduction for the acceptance of radio frequency electromagnetic field communication technologies.

## 1. Background

### 1.1. Introduction

Over the years, the services offered by wireless networks have modified our daily life. At first quickly adopted for their portability, mobile phones have become an indispensable tool in daily life. Wireless networks have increased enormously in recent years, and this trend is expected to continue [1]. Nowadays, base stations, smartphones, tablets, and WiFi routers are found nearly everywhere. The electromagnetic fields (EMFs) emitted by their antennas depend on the power emitted, and their amplitudes are inversely proportional to the distance of the antennas (the sources). However, such radio frequency electromagnetic fields (RF EMFs) also depend on the directivity and gain of the antennas that are focusing the signal toward areas of interest. Therefore, RF EMF human exposure is difficult to assess intuitively. Although the distance to the antenna can be estimated visually, the power emitted, the directivity and the frequency are not perceptible by human senses. It is, therefore, not scientific facts, such as RF EMF exposure levels [2,3], but rather, intuitive beliefs are crucial to a non-expert’s risk perception, as eloquently expressed by Peter Sandman years ago: “*The risks that kill you are not necessarily the risks that anger and frighten you*” [4]. Consequently, exploring the construct of intuitive risk perception has been a research focus for many years.

From a utilitarian view, both perceived risks and perceived benefits determine the preferences for options [5]) in general and, in particular, the acceptance of technologies [6]. However, because risk issues play a dominant role in public debates about modern technologies—such as wireless communication—social science research has focused on risk perceptions. Despite, or perhaps because of, the large number of studies conducted in this area, definitions of risk perception vary to a considerable degree. A look at the questionnaire items with which risk perception is assessed reveals the broadness of what is conceptualized as risk perception. Some use “worry” or “concern” interchangeably with risk perception, or see both as a single dimension of risk perception (see feelings-of-risk, [7,8]), while others have argued for a separation of these concepts [9]. Some measures could be termed as cognitive, while others involve stronger emotional cues. There have also been attempts to combine affective and cognitive items in the same scale (e.g., [8,10]).

In the current study, we focus on something that—in our view—most questionnaire-based risk perception studies have in common, i.e., that researchers measure risk perception with one or more rather abstract, closed questions. For instance, in the Eurobarometer study from 2010 [11], participants were asked “*How concerned are you about the potential health risks of electromagnetic fields?*” on a 4–point Likert scale ranging from “*not at all concerned*” to “*very concerned*”. According to this assessment, 46% of European citizens are “*fairly*” or “*very concerned*” about the potential health risks of electromagnetic fields (EMFs), while 51% are “*not very concerned*” or “*not at all concerned*”. However, this approach can be criticized both on the grounds of survey methodology in general (see [12] for a review) and risk perception measurement specifically [13,14]. Comparing closed question measurements of risk perception with qualitative data, Zwick [13] and Gaskell [14] both argue that Likert–scale measurements overestimate the “true” level of perceived risk. According to [13], this is especially a problem with technical and environmental risks. As a remedy, he suggests measuring the subjective importance of a variety of risks at the beginning of a risk perception questionnaire, so that a comparative evaluation of technological, social, environmental and everyday life hazards, becomes possible. The same problem has also been identified regarding the “worry” of becoming the victim of a crime [15]. Here, the authors compared closed questions with a question about the frequency with which people worried about a crime in the past year. The result was that asking a question about the frequency of a crime resulted in lower levels of worry being reported, when compared to responses to the closed question.

This finding regarding the fear of crime might also extend to other areas of risk. Some respondents expressing high concerns on closed survey questions may also be concerned in their daily life. In contrast, for other subjects expressing high concerns, the risk might not be relevant in everyday life.

The current study aims at extending the existing literature by transferring this latter idea to another risk area, namely the potential health risks of radio frequency electromagnetic fields (RF EMFs). The approach is similar to that of Gray [15], however, in contrast to that study, we do not only compare the closed question with a frequency question. In line with the ideas of the Austrian sociologist Alfred Schütz on the allocation of attention in everyday life [16,17] we suggest two theoretical concepts—thematic and discursive relevance—highlighting the issue of the *endurance* of risk-related concerns. Schütz coined the concept “thematic relevance” in the 1930s when he developed his theory of relevance structures. It refers to the emergence of themes in the human consciousness. In other words, it defines the object of attention. Schütz´ concept is of special importance for risk perception research. Here, the question is whether the risks that are asked in questionnaires are of relevance for the respondents. Previous risk perception research demonstrated remarkable differences between imposed and intrinsic risk relevance. For instance, [18] analyzed what risks people perceive and assess in their daily activities. It revealed that answers to simultaneous risk questions (i.e., made at the time the risk was experienced) differed from those that were retrospective or generic risk questions. In addition to thematic relevance, we introduce the concept of discursive relevance. It describes the communicative importance of a topic in everyday discussion. Risk research has only recently taken this perspective into account [19]. In the following study, thematic relevance describes how often someone usually thinks about a risk in a given period of time, for instance, during the last two weeks. Discursive relevance goes even further, capturing how often respondents discuss a risk with other people.

Based on these theoretical considerations, one could differentiate between at least four groups concerning RF EMF risk potentials: (1) subjects with low levels of concern and low thematic and discursive relevance; (2) subjects with high levels of concern but low thematic and low discursive relevance; (3) subjects with high levels of concern and high thematic relevance and low discursive relevance; and (4) subjects with high levels of concern, high thematic and high discursive relevance. Theoretically, however, other combinations are also possible.

### 1.2. Research Aims

The aim of the study is to test the feasibility and utility of this new approach to risk perception, that introduces thematic and discursive relevance as additional components, in order to make more sound extrapolations from questionnaire-based risk perception studies to real world conditions. In other words, the question is whether our new approach overcomes restrictions of the risk perception methodology that limit the ecological validity of its findings [20].

The following hypotheses, with respect to the aforementioned risk perception groups, were studied:

1. The suggested grouping of RF EMF risk perceptions according to three components—concern, thematic relevance, and discursive relevance—results in a meaningful distribution of responses.
2. People with high levels of concerns for whom RF EMF risk potentials are thematically or both thematically and discursively relevant, will evaluate RF EMF exposure situations differently in comparison people with high levels of concerns but with low levels of thematic and discursive relevance. They will express stronger negative feelings and moral concerns and view themselves as strongly exposed.
3. The effect of a reduction in RF EMF exposure on the acceptance of base stations depends on the thematic and discursive relevance of a respondent’s RF EMF risk perception.

The first hypothesis is based on the work of Alfred Schütz, as mentioned above. To our knowledge, however, there is currently no empirical evidence that can be used for specifying our assumptions about the structure of our three-component model of risk perception. Our expectation is that the emerging risk perception groups do not violate logical consistency. For instance, we expect the proportion of participants that are not and less concerned about RF EMF risk potentials, but think and talk about potential health effects of EMF, to be low. Furthermore, we expect that there are no participants who indicate that they talk about the issue, but do not think about it.

With respect to the second hypothesis, we follow the approach of one of our recent studies [21] that investigated the cognitive, affective, and moral evaluations of RF EMF exposure situations. Here, the cognitive evaluation focused especially on exposure issues. The third hypothesis is derived from a study conducted by Wiedemann [22]. Here, the level of risk acceptance was operationalized by the required distance from the exposure source (in this case, high voltage power lines) to one´s own home. This operationalization is based on the Lindell and Earle study [23] on risk perception of industrial facilities. They asked their respondents for the minimum safe distance that they would be willing to live from various hazardous facilities. Furthermore, Freudenstein [21] also used this approach to explore the impact of fictional exposure reduction scenarios on the acceptance of base stations.

## 2. Methods

The presented survey was conducted in August 2014 by the professional survey company SSI in seven European countries. Participation was voluntary. Data was evaluated anonymously. The online study had a total of 2454 participants, with 1809 respondents remaining after quality control (German sample *n* = 274, French sample *n =* 243, Spanish sample *n =* 241, Portuguese sample *n* = 290, Romanian sample *n =* 276, Serbian sample *n =* 291, and UK sample *n =* 194). The age distribution was balanced with a mean age of approx. 40 years, with 49.1% male and 50.9% female. The mean education of the respondents was 15.2 years. Regarding employment, most of the respondents were in paid work (57%, including employees, self-employed, and working for the family business), 11.3% of the respondents were unemployed and actively looking for a job, and 9.0% were studying.

The surveys—consisting of 33 questions—started with a short introduction describing the topic and the background of the study, as well as the confidential handling of their personal information. The background information was presented as: “*The background of the survey is the project LExNet: Low EMF Exposure Future Networks. Seventeen leading telecommunication operators, vendors, research centres and academic institutions from the EU cooperate in LExNet throughout 10 European countries. The reduction of exposure to radio frequency electromagnetic fields is examined and it is analysed technically in the project as well as how this will be accepted by the user*”. Before respondents answered the first question, some additional information on RF EMFs was presented: “*FOR YOUR INFORMATION: Electromagnetic fields (EMF) are produced and emitted by electrical devices. Mobile phones and base stations use EMF for transmission of voice and data.*”

All questions were translated into languages of the participating countries (German, French, Spanish, Portuguese, Dutch, Romanian, Montenegrin, Serbian) and double-checked with re-translation back into English. Demographic, political, and economic background-related items were partially adapted from the survey platform “European Social Survey” [24]. The dataset used and analyzed during the current study is available from the corresponding author on reasonable request.

For a detailed analysis, three groups were formed according to the subjects’ responses regarding the components of RF EMF risk perception, i.e., concern, thematic relevance, and discursive relevance (see Table 1).

The grouping of the study subjects into one of the three groups is based on the following assignment: (1) Not and less concerned group: subjects who scored ≤3 on the 5–point Likert scale for concerns, as well as for thematic and discursive relevance (*n =* 931); (2) not enduringly concerned: subjects that responded with 4 *=* “fairly concerned” and 5 *=* “very concerned” regarding concerns about the potential health effects of electromagnetic fields, but ≤3 on questions regarding relevance (*n =* 548); (3) enduringly concerned: subjects who scored ≥4 for concerns and 4 *=* “often” or 5 *=* “very often” on thematic relevance (*n =* 228). Due to the small of number of subjects who are concerned, and for whom RF EMF risks are thematically and discursively relevant, we have abstained from building a further group.

To measure the respondents’ personal perceived daily RF EMF exposure, a simple item was created and asked at the beginning of the questionnaire: “Think about your daily life, to which degree do you think you are exposed to electromagnetic fields from electronic devices (like mobile phones, WiFi router) and base stations?”, using a 5–point Likert scale from 1 = “Not at all”, to 5 = “To a very high degree”. Regarding intuitive exposure assessments, respondents had to respond as to how much they agree with four different statements on EMF exposure issues (1) “No matter how low the EMF exposure is, there is still a risk due to the fact that even a minimal exposure may result in negative health impacts”; (2) “Man-made electromagnetic fields are more dangerous than natural ones”; (3) “The deployment of base stations in residential areas is not a mere technical question, but one that should respect the views of the concerned citizen” and (4) “No matter whether or not I am exposed to EMF radiation, base stations simply scare me.” All of these statements were assessed on a 5–point Likert scale from 1 = “Not at all”, to 5 = “Absolutely”.

For an improved measurement of RF EMF exposure perception, as well as affective or moral evaluations of particular exposure situations, picture-guided scenarios were used. Every item included a picture where the exposure situations were displayed. The questionnaire contained several exposure scenarios presented in randomized order; however, the focus of the present analysis is on mobile phones and base stations. The relevant exposure scenarios used were (1) a woman using a cell phone, and (2) living in the vicinity of an antenna on the rooftop of the building next door that could be seen from a window in an everyday situation. The following questions then had to be answered. For affective evaluation: “Imagine you are the person depicted in the picture/living close to the building with the antennas, what kind of feelings about exposure would you have in this situation?” on a 5–point Likert scale from 1 = “Very positive”, to 5 = “Very negative”. For the moral evaluation: “In your opinion, does the situation depicted by the picture elicit any moral concerns about exposure?” on a 5–point Likert scale from 1 = “Not at all”, to 5= “Yes absolutely”. The subjective exposure perception was operationalized by: “In your opinion, how strong is the exposure to the person in the above picture/for a person living close to the building with antennas?” on a 5–point Likert scale from 1 = “Low”, to 5 = “High”. Further descriptions of this approach can be found in our previous papers ([21,25]). Testing the effects of exposure reduction on the acceptance of RF EMF, subjects were asked about their willingness to accept a base station deployment close to their homes in fictional exposure reduction scenarios. The distance between their homes and a base station (in m) was used to operationalize the acceptance of a base station, evaluated in four scenarios: Scenario 1 (S1): 0% exposure reduction, S2: 30% exposure reduction, S3: 50% exposure reduction, and S4: 70% exposure reduction, compared to the current level. (Question: “Roughly at what distance (m) would you accept a base station close to your home?”. “...if the exposure was reduced by 30%?”, “...if the exposure was reduced by 50%?”, “...if the exposure was reduced by 70%?”). Further details are provided elsewhere [21]. All analytical calculations were conducted using IBM, SPSS^®^, V20 (IBM, Armonk, NY, USA).

## 3. Results

### 3.1. Risk Perception Groups and Socio Demographic Patterns

**Figure 1:**
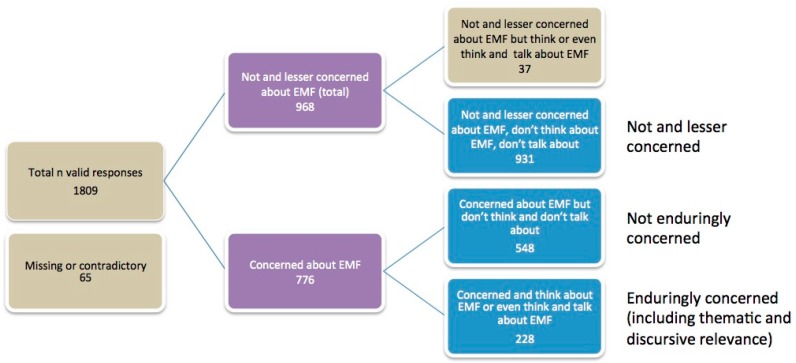
Distribution tree of the study participants regarding electromagnetic field (EMF) risk perception in terms of concern, thematic and discursive relevance. In each box, the number of included subjects is given.

The grouping of the respondents into the three aforementioned clusters showed the following distribution. Only about one-third (228) of respondents who claimed that they are concerned are enduringly concerned, i.e., report that they think, or think and talk about RF EMF risks in their everyday lives, while 548 are not enduringly concerned, and 931 are not and less concerned. The resulting distribution tree is displayed in Figure 1. A total of 102 respondents were excluded due to being not and less concerned yet still thinking about the topic (*n =* 37), as well as the interviewees with missing answers in one of the three questions responsible for grouping or with contradictory responses (talking about EMF topic but not thinking about it) (*n =* 65).

First, the three risk perception groups were compared with regard to differences in socio-demographic patterns using age, gender, education and living area as independent variables in a Welch’s ANOVA. Significant differences appeared only with respect to gender (Welch’s F(2, 590.12) = 7.44, *p =* 0.001). A subsequent Games–Howell post hoc test revealed differences comparing not and less concerned subjects with not enduringly concerned subjects (*p =* 0.001) and comparing not and less concerned and enduringly concerned respondents (*p =* 0.033). The proportion of men was significantly higher in the not and less concerned group.

### 3.2. Intuitive Exposure Assessments

The three risk perception groups were compared with respect to the perceived level of daily RF EMF exposure (on a 5–point Likert scale from 1 “Not at all” to “5 “To a very high degree”). The comparison between the groups with various manifestations of risk perception showed means of 3.41 for the not and less concerned group, 3.85 for the not enduringly concerned group and the highest mean for the enduringly concerned group, reaching 4.14. Statistical analysis by a Welch’s ANOVA revealed that the level of exposure perception differed significantly for each of the different groups, (Welch’s F(2, 659.30) = 74.59, *p* < 0.001). Games–Howell post hoc revealed significant differences between all groups (*p* < 0.001). Distribution for the frequency of given answers per group is shown in Table 2.

**Figure 2:**
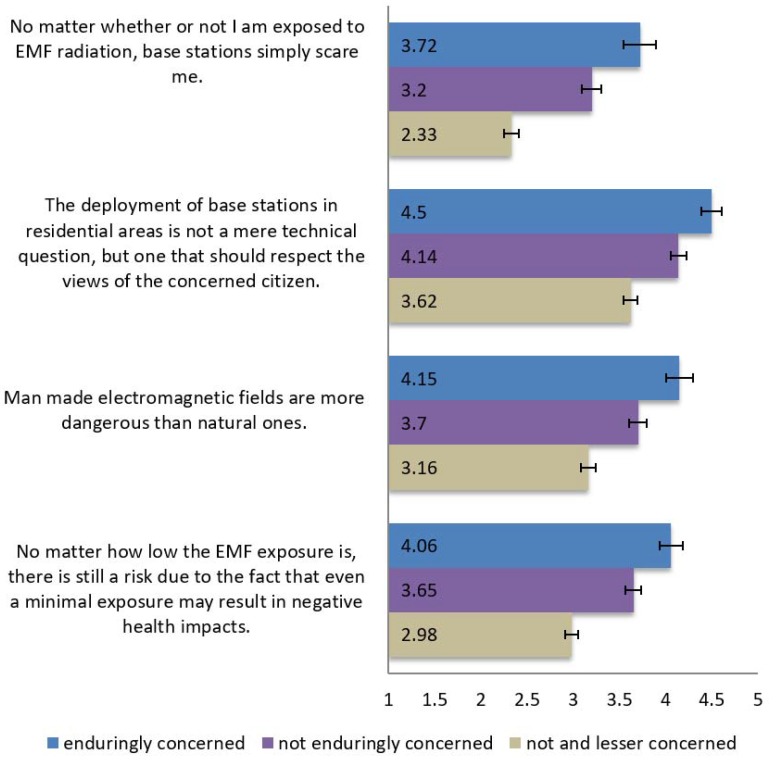
Beliefs about radio frequency electromagnetic field (RF EMF) exposure (Question: “Please tell us to what extent you agree with the following statements”; on a 5–point Likert scale from 1 = “Not at all” to 5 = “Absolutely”); statements and related means of the responses are indicated in the figure. Error bars indicate 95% CI.

Differences between the three risk perception groups were explored regarding their intuitive exposure assessments. Four issues were focused on: (1) a dose–response issue saying that any RF EMF exposure, regardless of the amount, causes detrimental health effects; (2) a nature vs. technology issue, where man-made RF EMF is considered more harmful than natural RF EMFs; (3) a location issue, i.e., selecting the location of base stations should take into account the acceptance of the residents; and (4) a fundamentalist exposure belief, saying that the base stations are just scary. A separate Welch´s robust test of equality of means indicated significant differences between the three groups regarding all four issues (issue 1: F(2, 629.44) = 133.48, *p* < 0.001; issue 2: F(2, 631.74) = 83.76, *p* < 0.001; issue 3: F(2, 682) = 92.61, *p* < 0.001; issue 4: F(2, 585.25) = 154.87, *p* < 0.001). Games–Howell post hoc tests revealed that the differences between the means of all three risk perception groups for all four issues were statistically significant (results are displayed in Figure 2, means are indicated).

### 3.3. Affective, Moral and Subjective Exposure Evaluations in Risk Perception Groups

This section analyzes how people from the three risk perception groups evaluate RF EMF exposure from base stations and mobile phones in fictional exposure situations. For both scenarios, we asked the subjects for their affective and moral evaluation of the RF EMF exposure situation, as well as their subjective exposure evaluations of the particular situations. Differences in group means are displayed in Table 3.

A consistent picture emerged. As in each case, the enduringly concerned group had the highest means for affective (mean_MP(Mobile_phone)_ = 3.46, mean_BS(Base_station)_ = 4.05) and moral (mean_M*P*_ = 3.80, mean_BS_ = 4.47) evaluations. Furthermore, the subjective evaluation of exposure strength (mean_M*P*_ = 4.08, mean_BS_ = 4.63) was consistently the highest for respondents with enduring concerns of potential EMF health risks. An analysis of variances using the three predictor variables of RF EMF risk perception (affective, moral, and exposure perception) as dependent variables was calculated to differentiate between the risk perception groups using the Welch Test. The results demonstrated statistically significant differences between the groups for affective, moral and exposure perception. Welch’s F values are indicated in Table 3, with highly significant differences (*p* < 0.001) across all evaluations. Games–Howell post hoc test also revealed significant results between all groups.

### 3.4. Differential Effects of RF EMF Exposure Reduction on the Acceptance of Base Stations in Risk Perception Groups

The effects of RF EMF exposure reduction on the acceptance of a base station were investigated for the three different risk perception groups. A general linear model with repeated measures was calculated using the three risk perception groups as between subjects factor, and the four exposure reduction scenarios as within subjects factor (exposure is reduced by 0%, 30%, 50%, and 70%). The acceptable distance between a base station and the respondent’s home was used as the dependent variable. The results showed a significant main effect for the repeated factor exposure scenario (F(1.42, 2173.55) = 360.22, *p* < 0.001 using Greenhouse–Geisser, η^2^ = 0.190), as well as for the between-subject effect for risk perception groups (F(2, 1536) = 31.26, *p* < 0.001, η^2^ = 0.039). The interaction between the exposure reduction scenario and the non-repeated factor risk perception group also showed a significant difference: (F(2.83, 2173.55) = 17.08 and *p* < 0.001, using Greenhouse–Geisser, η^2^ = 0.022).

**Figure 3:**
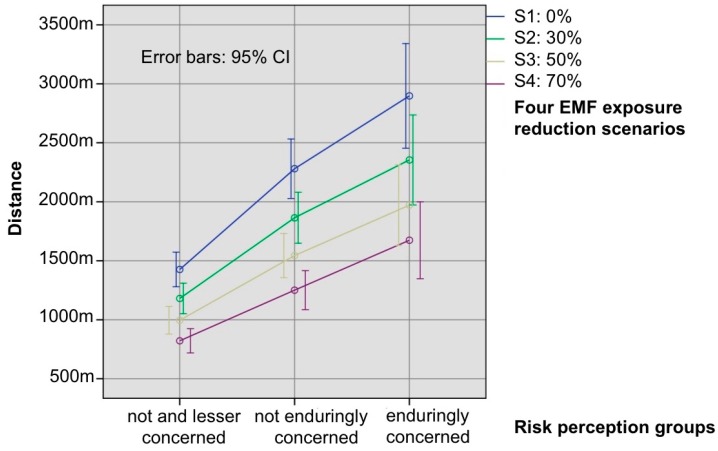
Effect of a reduction in RF EMF exposure on the acceptance of base stations. Provided are the means of requested distances to the next base station, as a function of the risk perception groups. Acceptance measured by the question: “Roughly at what distance (m) would you accept a base station close to your home, if the exposure was reduced by 0%/30%/50%/70%?“. Meaning of lines: blue = 0% exposure reduction, green = 30% exposure reduction, yellow = 50% exposure reduction, violet = 70% exposure reduction. Respondents with distances higher than 10,000 m are excluded. Error bars indicate 95% CI.

These results indicate that the factors “exposure reduction” and “risk perception group” have a statistically significant influence on the accepted distance from the base station, and that the impact of the exposure reduction on this distance depends on group membership. The highest impact was found for the enduringly concerned group, where the mean required distance to the base station was reduced from 2897 m at 0% exposure reduction, to 1674 m for 70% reduction. The mean distance for the not enduringly concerned group decreased from 2280 m to 1251 m, and for the not and less concerned group from 1426 m to 821 m, comparing the exposure reduction scenarios 0% and 70% (see Figure 3).

Table 4 indicates Games–Howell post-hoc tests for differences of accepted distances to the base station between the three risk perception groups for every single exposure reduction scenario. Significant differences were found between the not and less concerned group and not enduringly concerned group (*p* < 0.001 for all comparisons), as well as for the not and less concerned group compared to the enduringly concerned group (*p* < 0.001 for all comparisons). There were no significant differences between the enduringly concerned group and the not enduringly concerned group (from *p =* 0.50 to *p =* 0.80).

## 4. Discussion

The presented study demonstrates that risk perception should not only be differentiated according to the level and range of concerns, but also in terms of the endurance of these concerns. It should not only be distinguished between people with high and low levels of concern, but also between those with enduring concerns and concerns that are only expressed in a survey situation. Survey methodologists (see Schwarz [12]) have already underlined that the questionnaire—particularly the question wording and question format—can strongly influence the obtained findings. This could be also a problem for risk perception research. If taken seriously, it threatens the validity of conventional risk perception surveys, e.g., the Eurobarometer survey on EMF risk perception [11].

In line with this thinking, the present study focused especially on the distinction between not enduringly concerned and enduringly concerned people. Both groups expressed high risk perception in the survey, but only the latter reported that RF EMF risk was a topic which they thought about in their daily life. Enduringly concerned people showed a number of unique features: they believed that they are highly exposed to RF EMFs and revealed a more negative anthropocentric view, being convinced that man-made EMFs are more dangerous than natural EMFs. Of course, full exploration of how this effect occurs will require further consideration of the knowledge-base on which these questions are answered. This applies in particular to the distinction between natural and man-made RF EMF. A preliminary explanation could be given by Rozin’s [26] suggestion that nature implies safety for most people.

In addition, they also displayed a less elaborated concept of how RF EMF exposure impacts health: enduringly concerned people were more convinced that even a very low RF EMF exposure can have detrimental health effects when they were asked directly by a closed question. Even if enduringly concerned subjects are sensitive to exposure reduction, this group does not appear to comply with the basic principle that “the dose makes the poison”, which dates back to Paracelsus. In addition, this group has a stronger dogmatic view on base stations being perceived as ‘scary’ objects, no matter whether the person is exposed to RF EMF from this source or not. Compared to the other groups, the enduringly concerned respondents also considered the location of base stations as more of a political issue, that furthermore should take into account the views of residents who might be affected by the location.

The in-depth analysis of RF EMF risk perception revealed that the group of enduringly concerned subjects differed from the other groups regarding how their risk perception was linked to feelings, moral aspects, and perceived exposure. This particular group had higher estimates of RF EMF exposure regarding both the use of mobile phones, and living in the vicinity of base stations. They regarded EMF exposure as a moral issue, as well as a situation that elicits negative feelings.

Moreover, it is surprising that the group of enduringly concerned subjects was sensitive to exposure reduction. The acceptance of base stations in their own neighborhood depended on the amount of RF EMF exposure reduction. Compared to the not and less concerned group, the not enduringly concerned/enduringly concerned group had a higher response to exposure reduction; however, the requested mean distance for accepting a base station still by far exceeded the distances deemed safe by the exposure guidelines. For instance, a 70% exposure reduction, compared to the current levels, reduces the average requested distance in the enduringly concerned group from 2897 m to 1674 m. Regarding the overall exposure, it has to be pointed out that the belief of being less exposed to RF EMFs when base stations are far away, albeit common among the lay population [27,28], is likely to be erroneous under most circumstances, for those people that own and use a mobile phone. As Claassen [29] has shown empirically, a better understanding of the distance–exposure relationship leads to lower risk perceptions and increases the acceptance of public EMF sources in people´s neighborhoods. Future research could usefully investigate whether this is equally observable among enduringly concerned and not enduringly concerned people.

The findings clearly support the notion that risk perception studies should take into account the issue of extrapolation from questionnaire situations to everyday life. As a first step towards this aim, our conceptualization of risk perception studies as a three-component approach appears to be promising. To focus on concerns alone leaves risk perception studies incomplete; one deals more adequately with risk perception when the decisive role of thematic and discursive relevance is taken into account.

Finally, some limitations of our findings should be taken into account. First, we only considered personal risks. Risk to other persons cannot be commented on here. A second issue refers to categorization into the risk perception groups. There were no natural cutoff points between levels of concern, levels of thematic relevance, and levels of discursive relevance. Therefore, our results may vary according to the chosen cutting points. Another related issue is a potential effect of the order of questions relating to the three aforementioned aspects of risk perception. Currently, we have no knowledge concerning this. Further research is needed to fill this knowledge gap.

A further problem that deserves clarification refers to the operationalization of the endurance of risk perception. Here, the related question was “How often in your daily life do you think about the topic “potential health effects of electromagnetic fields?”. This is open to considerable interpretation, and future studies could usefully explore the relevance of concerns in daily life using more definite frames of reference, such as by providing specific time spans, or measuring concern longitudinally.

## 5. Conclusions

The conventional measurement of risk perception can result in inaccurate estimations of public concerns, and in doing so, trigger exaggerated views about the share of the population who are in opposition to RF EMF-based communication technologies.

Our findings indicate that it makes sense to determine the degree to which RF EMF risk perceptions are relevant in everyday life. It seems that the group of enduringly concerned subjects has more affective and morally shaped concerns. This characteristic should be taken into account in risk communication. Furthermore, these people strongly believe that even a small amount of RF EMF exposure may result in health risks. It can also be concluded that RF EMF exposure reduction is an argument that is relevant for those that are enduringly concerned. Exposure reduction is an effective route for RF EMF risk management that can improve the acceptance of base stations; however, the problem remains that agreeing with the demands of the enduringly concerned group—the required distance of the base stations to their homes—would mean that, at least in urban areas, the architecture of the networks for wireless communication would face potentially unworkable challenges.

## Figures and Tables

**Table 1:** Questions on risk perception of electromagnetic fields (EMFs) in general and thematic and discursive relevance.

Question	Answer Option
Concerns	5–point Likert scale from 1 = not at all concerned, 2 = not very concerned, 3 = neither nor, 4 = fairly concerned, 5 = very concerned
“*How concerned are you about the potential health effects of electromagnetic fields in general*?”	
Thematic relevance	5–point Likert scale from 1 = never, 2 = not very often, 3 = sometimes, 4 = often, 5 = very often
“*How often in your daily life do you think about the topic “potential health effects of electromagnetic fields”?*”	
Discursive relevance	5–point Likert scale, same as for above
*“How often in your daily life do you talk about potential health effects of EMF with other people (including conversation, via Facebook, twitter, chat, online forum or similar)?*”	

**Table 2:** Perceived level of daily radio frequency electromagnetic field (RF EMF) exposure per group (on a 5–point Likert scale from 1 “Not at all” to “5 “To a very high degree”) across the different manifestations of risk perception (Question: “Think about your daily life, to which degree do you think you are exposed to electromagnetic fields from electronic devices (such as mobile phones, WiFi router) and base stations?”).

Daily RF EMF Exposure	Number and % within Groups			Total
	Not and Lesser Concerned	Not Enduringly Concerned	Enduringly Concerned	
(1) Not at all	29 (3.1%)	2 (0.4%)	0 (0.0%)	31 (1.8%)
(2)	155 (16.7%)	38 (6.9%)	7 (3.1%)	200 (11.7%)
(3)	326 (35.1%)	156 (28.5%)	45 (19.8%)	527 (30.9%)
(4)	246 (26.5%)	197 (35.9%)	81 (35.7%)	524 (30.8%)
(5) To a very high degree	173 (18.6%)	155 (28.3%)	94 (41.4%)	422 24.8%)
Total	929 (100%)	548 (100%)	227 (100%)	1704 (100%)

**Table 3:** Means and analysis of variances of affective and moral evaluation, subjective exposure perception of base stations and mobile phones per group (n/l con *=* not and less concerned; not end, co*n =* not enduringly concerned; endcon = enduringly concerned), on 5–point Likert scale from 1 = “Very positive”, to 5 = “Very negative” for affective evaluation; from 1 = “Not at all”, to 5 = “Yes absolutely” for moral evaluation; from 1 = “Low”, to 5 = “High” for exposure evaluation.

Evaluation of	N/l Con	Not End Con	End Con	Total Mean	*p*	F (Welch)
Mobile phone (MP) calls:						
Affective evaluation	2.86	3.09	3.46	3.02	<0.001	53.31
Moral evaluation	2.37	3.08	3.80	2.79	<0.001	154.67
Subjective exposure perception	2.92	3.63	4.08	3.31	<0.001	185.33
Base stations						
Affective evaluation	3.41	3.69	4.05	3.59	<0.001	31.22
Moral evaluation	3.21	3.94	4.47	3.62	<0.001	149.43
Subjective exposure perception	3.42	4.20	4.63	3.85	<0.001	131.98

**Table 4:** Games–Howell post hoc test for in between group differences among risk perception (RP) groups and accepted distances to a base station for various exposure reduction scenarios. (n/l con = not and less concerned; not end, con = not enduringly concerned; endcon *=* enduringly concerned).

Exposure Reduction Scenario	RP Groups	RP Groups	Mean Difference Distance (M)	*p*
0%	n/l con	not end con	872	<0.001
		end con	1471	<0.001
	not end con	n/l con	872	<0.001
		end con	600	0.055
	end con	n/l con	1471	<0.001
		not end con	600	0.055
30%	n/l con	not end con	684	<0.001
		end con	1174	<0.001
	not end con	n/l con	684	<0.001
		end con	490	0.072
	end con	n/l con	1174	<0.001
		not end con	490	0.072
50%	n/l con	not end con	559	<0.001
		end con	976	<0.001
	not end con	n/l con	549	<0.001
		end con	427	0.080
	end con	n/l con	976	<0.001
		not end con	427	0.080
70%	n/l con	not end con	429	<0.001
		end con	853	<0.001
	not end con	n/l con	429	<0.001
		end con	424	0.060
	end con	n/l con	853	<0.001
		not end con	424	0.060

## Availability of Data and Materials

The datasets used and analyzed during the current study available from the corresponding author on reasonable request.

## Abbreviations

ANOVA	Analysis of variance
BS	base station
End con	enduring concerned
MP	mobile phones
N/l con	not and less concerned
Not end con	not enduringly concerned
RF EMF	radio frequency electromagnetic field
RP	risk perception
S	Scenario
SPSS^®^	Statistical Package for the Social Sciences
